# Supplementary material for: Enabling high‐throughput biology with flexible open‐source automation
Source: Mol Syst Biol. 2021 Mar 25;17(3):e9942. doi: 10.15252/msb.20209942 (PMC7993322; doi:10.15252/msb.20209942)
Supplement: Supplementary file 2 — Appendix [file MSB-17-e9942-s002.docx]

# Appendix

**Enabling high-throughput biology with flexible open-source automation**

Emma J Chory^1,2,3^ *, Dana W Gretton^1,^*^,✝^ , Erika A DeBenedictis^1,4^, Kevin M Esvelt^1,#^

^1^Media Laboratory, Massachusetts Institute of Technology, Cambridge, MA 02139, USA

^2^Institute for Medical Engineering and Science, Massachusetts Institute of Technology, Cambridge, MA 02139, USA

^3^Broad Institute of MIT and Harvard, Cambridge, MA 02142, USA

^4^Department of Biological Engineering, Massachusetts Institute of Technology, Cambridge, MA 02139, USA

* Designates equal contribution

^✝^ Correspondence for software development

^#^ For all other correspondence: [esvelt@mit.edu](mailto:esvelt@mit.edu)

[Appendix 1](#_Toc63328408)

[Appendix Figures 2](#_Toc63328409)

[Appendix Figure S1: Layers of Abstraction in Pyhamilton 2](#_Toc63328410)

[Appendix Figure S2: Population Dynamics Diffusion Matrix. 3](#_Toc63328411)

[Appendix Figure S3: Turbidostat Controller Simulation 4](#_Toc63328412)

[Appendix Figure S4: High-throughput turbidostats code 5](#_Toc63328413)

[Appendix Figure S5: Turbidostat Controller Limitations in varying media. 6](#_Toc63328414)

[Appendix Figure S6: Simulations of Turbidostat Controller Limitations 7](#_Toc63328415)

# Appendix Figures

**
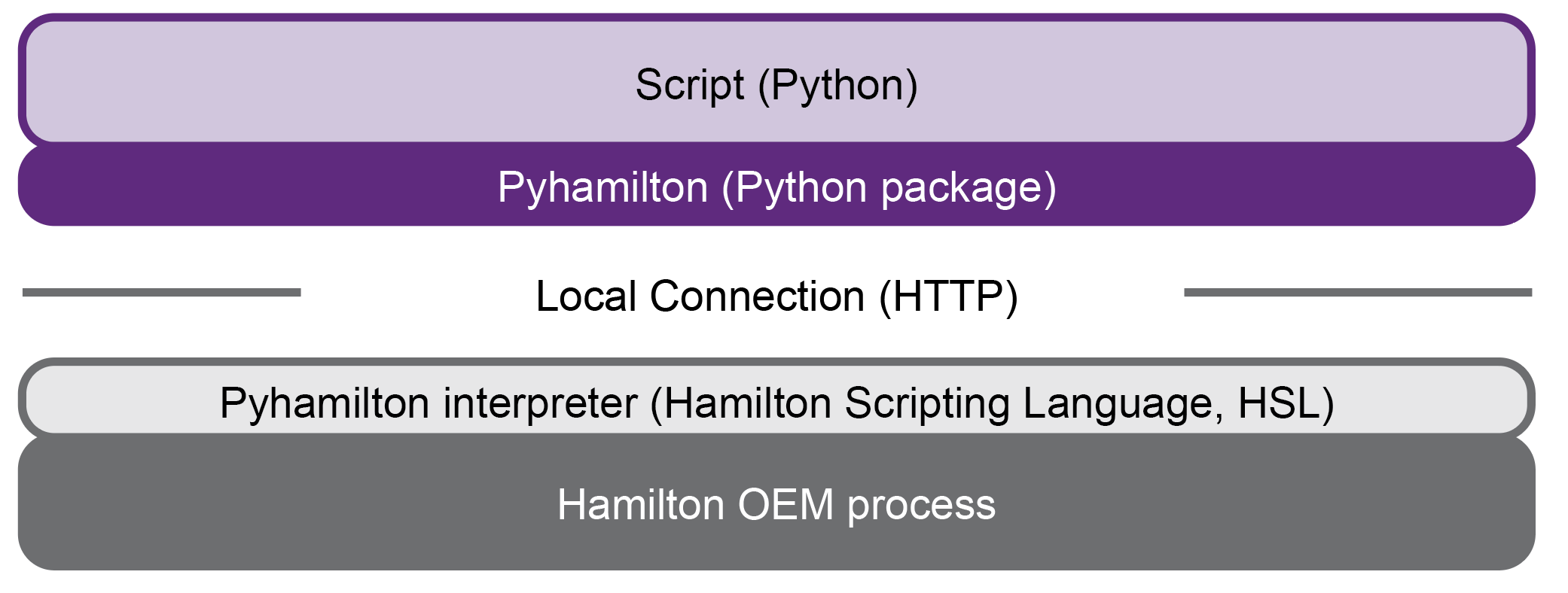
**

**
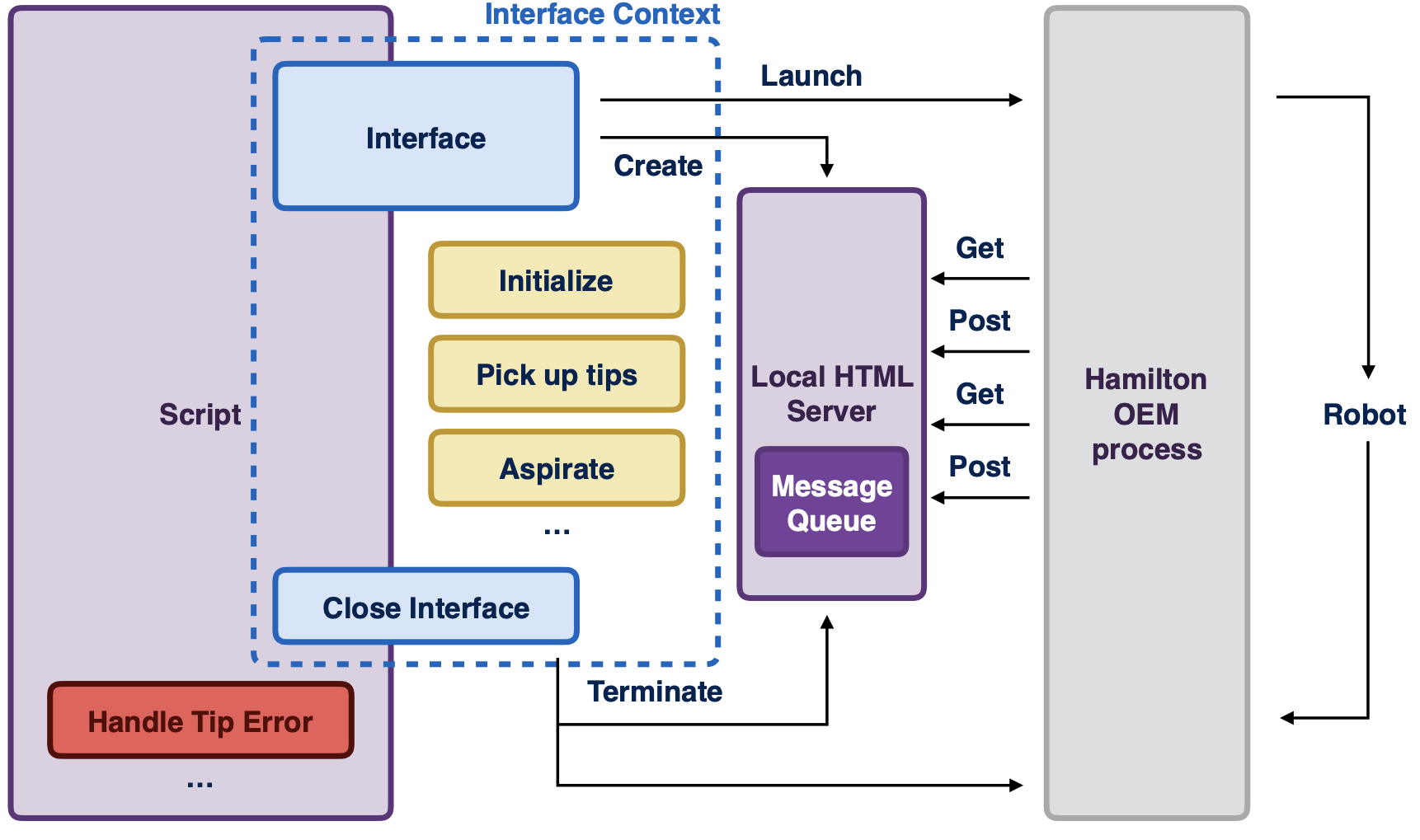
**

## Appendix Figure S1: Layers of Abstraction in Pyhamilton

Pyhamilton it uses a platform-independent, web-based protocol (HTTP) and common readable data format (JSON) to bridge Python and the Hamilton Scripting Language (HSL).


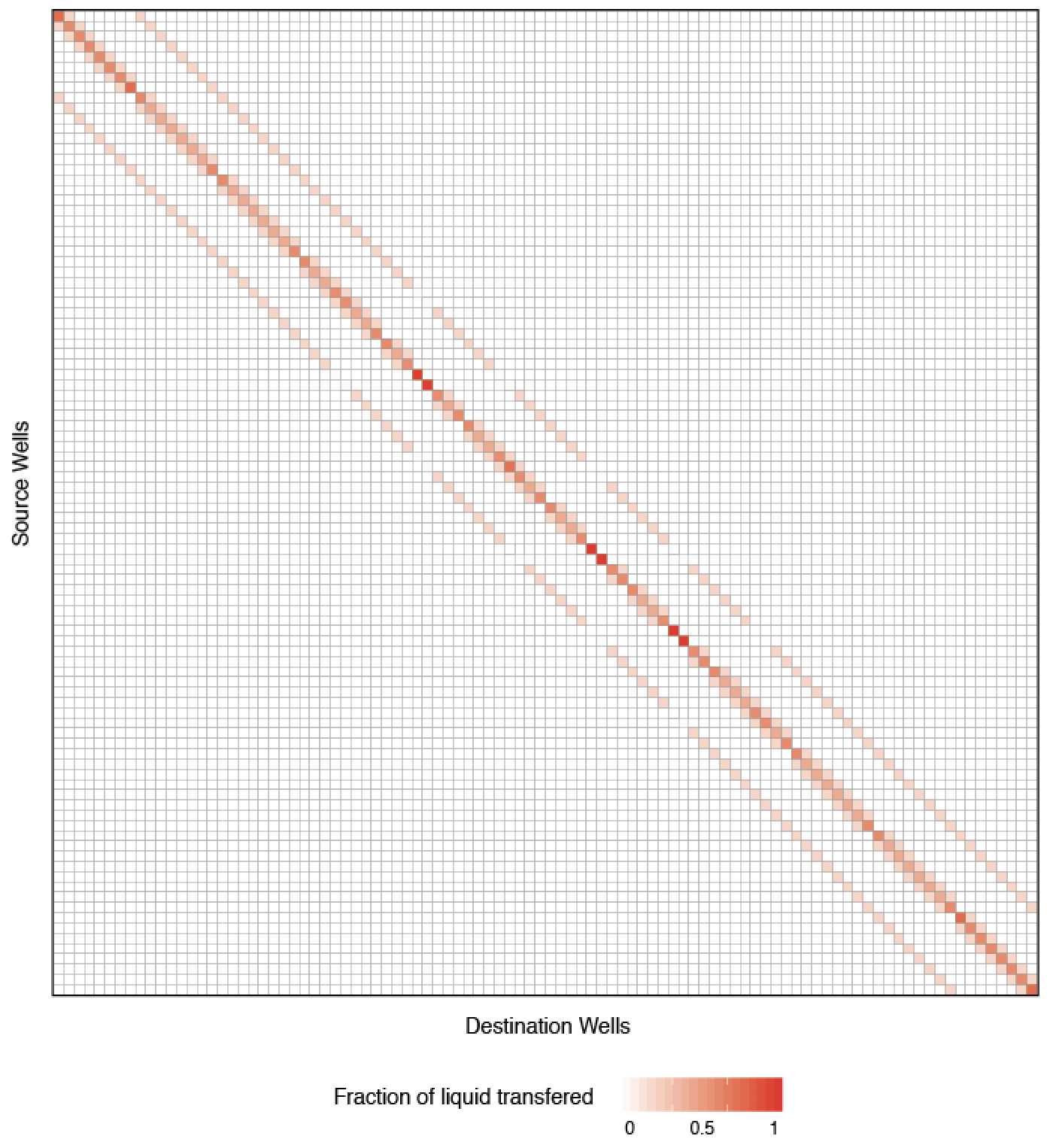


## Appendix Figure S2: Population Dynamics Diffusion Matrix.

Pyhamilton can be used in concert with all typical Python modules. The population dynamics application (Fig. 1D) makes use of matrix multiplication implemented by rectangular arrays from the scientific computing Python package NumPy. The diffusion matrix models arbitrary rates of flow from any of 96 wells in a microplate to all other wells. Gradient color scale represents fractional magnitudes of liquid transfers. Rows of the diffusion matrix sum to 1. Successive steps are computed by repeated multiplication of an initial 96-entry concentration vector by the 96x96 diffusion matrix. In this matrix, the dark main diagonal indicates that most of each population stays in its source well. Entries above and below the main diagonal by 1 and 8 rows represent transfers to vertical and horizontal neighbors respectively. All other entries are zero because population flow is not modeled to occur diagonally or beyond immediate neighbors. Obstacles to gene flow are captured by lighter colored areas in off-diagonal entries. Though this matrix is symmetric in that flows in both directions between each pair of wells are the same, matrix symmetry is not a requirement in general. The matrix construction facilitates offline analysis and visualization prior to robot execution. This construction would be difficult to implement in existing programming applications for Hamilton robots. Available online at: <https://github.com/dgretton/pyhamilton_population_dynamics/blob/master/flow_matrix.csv>.


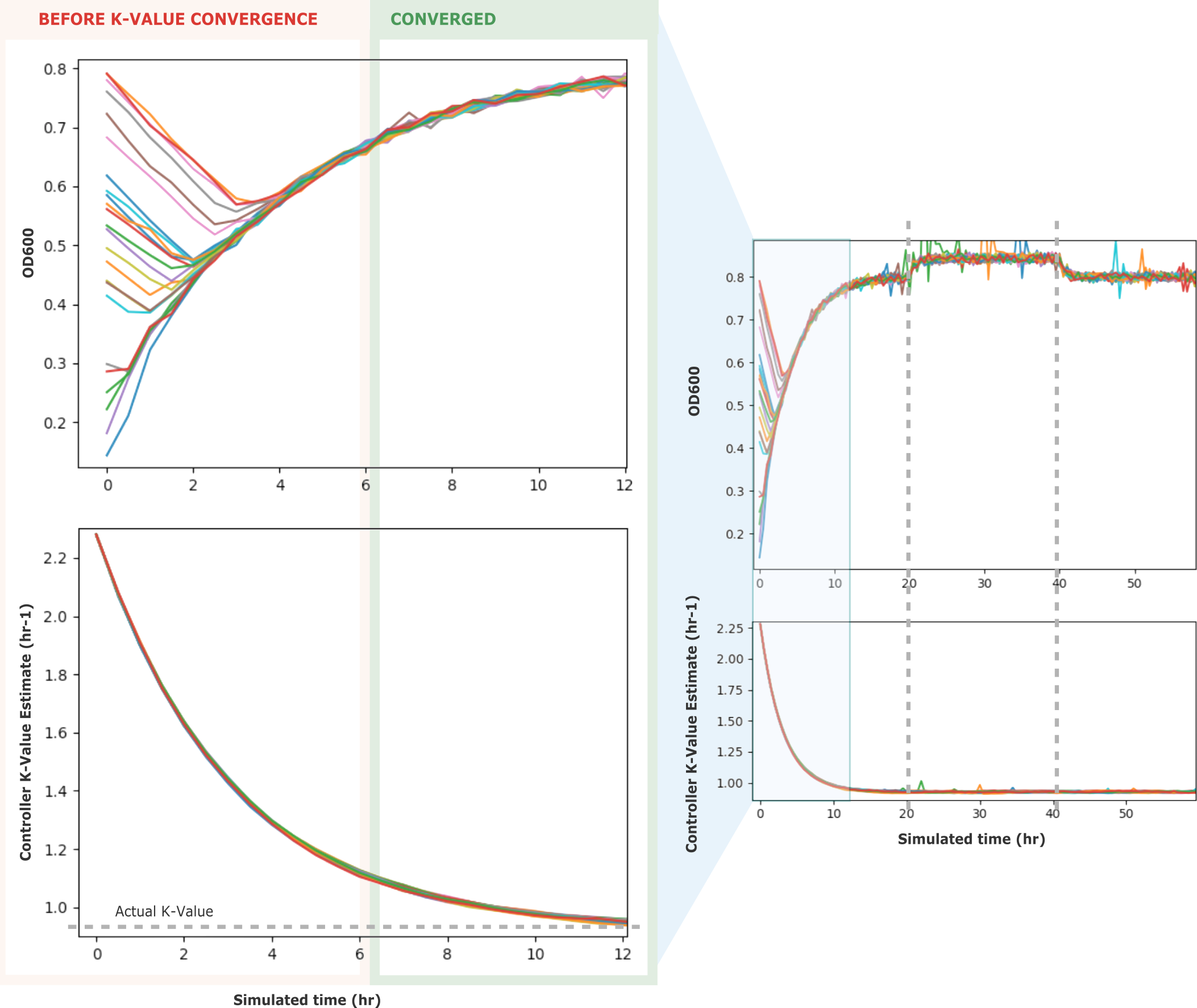


## Appendix Figure S3: Turbidostat Controller Simulation

24 simulated turbidostats using the same controllers as experiments with initial k-value estimate of 2.3 hr^-1^ (20-minute doubling time typical of E. coli in LB Media) and actual k-value 0.93 hr^-1^ (typical of metabolite-poor media) converge in 12 hours. Start conditions vary between OD 0.1 and OD 0.8. Simulation includes uniform pipetting volume noise model and power law measurement noise model (spurious peaks). Controllers initially over-replace media in higher density cultures due to growth rate overestimate, causing OD to drop temporarily, before recovering as the k-value estimate converges more closely to the actual k-value. This behavior is exactly recapitulated in the turbidostat convergence study (Fig. 2D), indicating good correspondence between model and system. The simulated turbidostat OD setpoint was adjusted ±0.1 at 20 hours and 40 hours. Though the OD measurements and the controller’s transfer volume commands both change at these times, the inferred k-value stays constant.

**# define number of turbidostats (4 plates)**

**num_turbs = 384**

**def main():**

**## define required labware
 labware = plates, tip_boxes, media_sources**

**## define plate reader protocols
 reader_protocols = ['absorbance', 'mCherry', 'YFP', 'CFP']
 while True: ## Maintain turbidostats indefinitely**

**## Service each plate of turbidostats (in loop)**

**for turbs_for_plate, controllers_for_plate, labware_for_plate in turbidostat_details:**

**## identify this plate’s materials
 plate, tips, media_supply = labware_for_plate**

**## Simultaneously read plate, and perform pipetting steps**

**platedatas = measure_plate(plate, reader_protocols, simultaneously_execute=service_prev_plate)
 ## save plate reader data
 record_readings(plate, turbs_for_plate, platedatas)**

**## Calculate bacterial OD from optical density calibration curve**

**od_readings = convert_to_ods(platedatas)**

**## calculate replacement volumes from transfer function**

**remember.replace_vols = transfer_function(controllers_for_plate, od_readings)**

**## set up pipetting steps for next plate**

**def service_prev_plate(args=(plate, tips, media_supply)):**

**service(remember.replace_vols, *args)**

**## load Hamilton and plate reader**

**with HamiltonInterface() as ham_int, ClarioStar() as reader_int:**

**## Define instruments used in experiment**

**sys_state.instruments = ham_int, reader_int**

**## initialize robot**

**system_initialize()**

**## Run method**

**main()**

##

## Appendix Figure S4: High-throughput turbidostats code


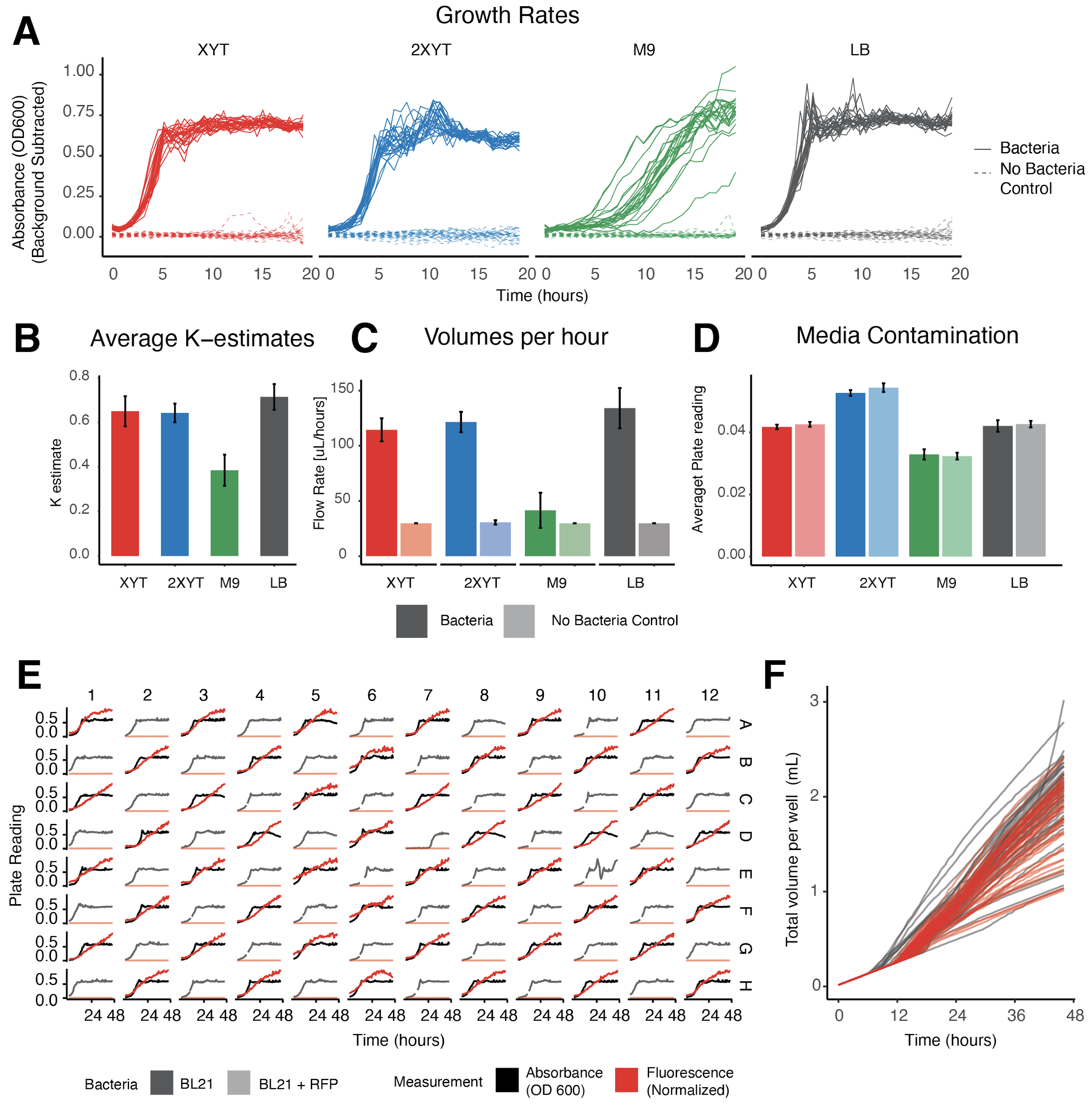


## Appendix Figure S5: Turbidostat Controller Limitations in varying media.

**(a)** Real-time absorbance measurements of 192 cultures (2x 96 well plates) grown in either M9, XYT, 2XY, or LB media, with no bacteria controls **(b)** Average K-estimate of each bacteria growth condition demonstrates that fast and slow growing strains can both be supported. **(c)** Average volumes per hour of media consumed by each growth condition demonstrates that a 2mL deep well plate can support growth of a high growing strain for >16 hours without user intervention (100 uL/hour per hour in exponential growth phase), while a slow growing strain can be maintained for over 30 hours without user intervention (~ 50uL per hour in exponential growth phase). **(d)** Media sources show no detectable back contamination after 24 hours without replenished media. **(e)** Absorbance (black) and Normalized fluorescence (red) BL21 Cultures (50% opacity) and BL21 cultures expressing RFP (100% opacity) grown over 48 hours. **(f)** Total amount of media consumed over 48 hours for cultures shown in (e).

**
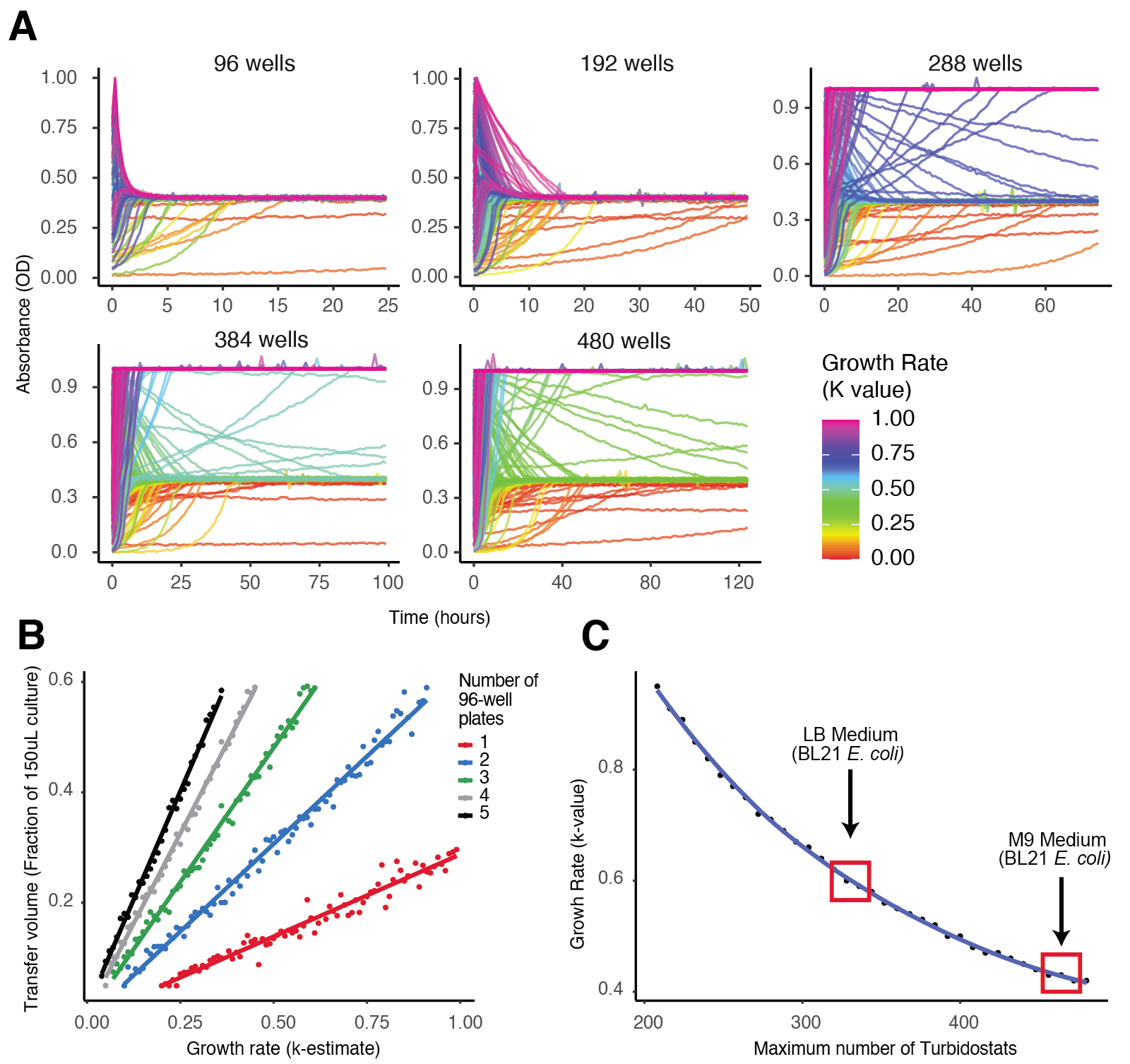
**

## Appendix Figure S6: Simulations of Turbidostat Controller Limitations

**(a)** Simulations of growth equilibration of bacteria with varying exponential growth constants (k-values, hr^-1^) when maintained at an OD setpoint of 0.4 in between 1-5 96-well plates. **(b)** Range of feasible transfer volumes as a function of growth rate when maintained in between 1-5 96-well plates. **(b)** Maximum number of turbidostats that can be maintained at a given growth rate. Average BL21 *E. coli* k-values when grown in either LB Medium or M9 medium are highlighted for reference.
