# Supplementary material for: Enabling high‐throughput biology with flexible open‐source automation
Source: Mol Syst Biol. 2021 Mar 25;17(3):e9942. doi: 10.15252/msb.20209942 (PMC7993322; doi:10.15252/msb.20209942)
Supplement: Supplementary file 5 — Dataset EV1 [file MSB-17-e9942-s009.pdf]

Pyhamilton

Contents

Module `pyhamilton` **4**
Sub-modules 4

Module `pyhamilton.deckresource` **4**
Classes 5
Class `DeckResource` 5
Descendants 5
Class variables 5
Methods 5
Class `LayoutManager` 6
Static methods 6
Methods 7
Class `Plate12` 8
Ancestors (in MRO) 8
Methods 8
Class `Plate24` 8
Ancestors (in MRO) 8
Methods 8
Class `Plate96` 9
Ancestors (in MRO) 9
Class `ResourceIterItem` 9
Descendants 9
Class `ResourceType` 9
Class `Standard96` 10
Ancestors (in MRO) 10
Descendants 10
Methods 10
Class `Tip` 10
Ancestors (in MRO) 10
Class `Tip96` 10
Ancestors (in MRO) 10
Class `Vessel` 11
Ancestors (in MRO) 11
Class variables 11
Methods 11

Module `pyhamilton.defaultcmds` **11**
Variables 11
Variable `defaults_by_cmd` 11

Module `pyhamilton.interface` **23**
Functions 23
Function `run_hamilton_process` 23
Classes 23
Class `HamiltonCmdTemplate` 23
Static methods 24

Methods 24
Class `HamiltonInterface` 24
Class variables 25
Methods 25

Module `pyhamilton.oemerr` **28**
Variables 28
Variable `HAMILTON_ERROR_MAP` 28
Classes 28
Class `AntiDropControlError` 28
Ancestors (in MRO) 28
Class `AreaAlreadyOccupiedError` 29
Ancestors (in MRO) 29
Class `BarcodeAlreadyUsedError` 29
Ancestors (in MRO) 29
Class `BarcodeError` 29
Ancestors (in MRO) 29
Class `BarcodeMaskError` 29
Ancestors (in MRO) 30
Class `BarcodeNotUniqueError` 30
Ancestors (in MRO) 30
Class `CalibrateError` 30
Ancestors (in MRO) 30
Class `ClotError` 30
Ancestors (in MRO) 30
Class `CoverOpenError` 31
Ancestors (in MRO) 31
Class `DecapperError` 31
Ancestors (in MRO) 31
Class `DecapperHandlingError` 31
Ancestors (in MRO) 31
Class `DelimiterError` 31
Ancestors (in MRO) 32
Class `ExecutionError` 32
Ancestors (in MRO) 32
Class `HamiltonDeckResourceError` 32
Ancestors (in MRO) 32
Descendants 32
Class `HamiltonError` 32
Ancestors (in MRO) 32
Descendants 33
Class `HamiltonInterfaceError` 33
Ancestors (in MRO) 33
Descendants 33
Class `HamiltonReturnParseError` 33
Ancestors (in MRO) 33
Class `HamiltonStepError` 33
Ancestors (in MRO) 33
Descendants 34
Class `HamiltonSyntaxError` 34
Ancestors (in MRO) 35
Class `HamiltonTimeoutError` 35
Ancestors (in MRO) 35
Class `HardwareError` 35
Ancestors (in MRO) 35
Class `IllegalInterventionError` 35
Ancestors (in MRO) 35
Class `IllegalTargetPlatePositionError` 36

Ancestors (in MRO) 36
Class ImpossibleToOccupyAreaError 36
Ancestors (in MRO) 36
Class ImproperAspirationOrDispenseError 36
Ancestors (in MRO) 36
Class ImproperDispensationError 36
Ancestors (in MRO) 37
Class InsufficientLiquidError 37
Ancestors (in MRO) 37
Class InvalidErrCodeError 37
Ancestors (in MRO) 37
Class KitLotExpiredError 37
Ancestors (in MRO) 37
Class LabwareError 38
Ancestors (in MRO) 38
Class LabwareGrippedError 38
Ancestors (in MRO) 38
Class LabwareLostError 38
Ancestors (in MRO) 38
Class LiquidLevelError 38
Ancestors (in MRO) 39
Class NoCarrierBarcodeError 39
Ancestors (in MRO) 39
Class NoCarrierError 39
Ancestors (in MRO) 39
Class NoLabwareError 39
Ancestors (in MRO) 39
Class NoTipError 40
Ancestors (in MRO) 40
Class NotAspiratedError 40
Ancestors (in MRO) 40
Class NotDetectedError 40
Ancestors (in MRO) 40
Class NotExecutedError 40
Ancestors (in MRO) 41
Class ParameterError 41
Ancestors (in MRO) 41
Class PositionError 41
Ancestors (in MRO) 41
Class PressureLLDError 41
Ancestors (in MRO) 41
Class ResourceUnavailableError 42
Ancestors (in MRO) 42
Class SlaveError 42
Ancestors (in MRO) 42
Class TADMOvershotError 42
Ancestors (in MRO) 42
Class TADMUndershotError 42
Ancestors (in MRO) 43
Class TemperatureError 43
Ancestors (in MRO) 43
Class TipPresentError 43
Ancestors (in MRO) 43
Class UnexpectedLabwareError 43
Ancestors (in MRO) 44
Class UnexpectedcLLDError 44
Ancestors (in MRO) 44
Class UnloadError 44

Ancestors (in MRO) . . . 44
Class `WashLiquidError` . . . 44
Ancestors (in MRO) . . . 44
Class `WrongCarrierError` . . . 45
Ancestors (in MRO) . . . 45
Class `WrongLabwareError` . . . 45
Ancestors (in MRO) . . . 45

Module `pyhamilton.util` **45**
Classes . . . 45
Class `ChannelHead` . . . 45
Descendants . . . 45
Methods . . . 45
Class `FlyTransfer` . . . 46
Ancestors (in MRO) . . . 46
Class `GroupableAction` . . . 46
Ancestors (in MRO) . . . 46
Descendants . . . 46
Class `HamiltonAction` . . . 46
Descendants . . . 46
Methods . . . 46
Class `HamiltonCoordinator` . . . 47
Methods . . . 47
Class `HamiltonDevice` . . . 47
Class `Independent8Channel` . . . 47
Ancestors (in MRO) . . . 47
Class `Mix` . . . 47
Ancestors (in MRO) . . . 47
Class `Standard96Channel` . . . 47
Ancestors (in MRO) . . . 48
Class `TipPickup` . . . 48
Ancestors (in MRO) . . . 48
Methods . . . 48
Class `Transfer` . . . 48
Ancestors (in MRO) . . . 48
Descendants . . . 48
Methods . . . 48

Module `pyhamilton`

Sub-modules

- pyhamilton.deckresource
- pyhamilton.defaultcmds
- pyhamilton.interface
- pyhamilton.oemerr
- pyhamilton.util

Module `pyhamilton.deckresource`

Couplings to Hamilton deck layouts.

Module pyhamilton.deckresource provides convenience classes and methods for interacting safely with Hamilton's Layout (.lay) files. It also implements transformations between well indexes and coordinates for a variety of labware, such as plates and tips.

Classes

Class `DeckResource`

```
class DeckResource(
    layout_name
)
```

Descendants

- pyhamilton.deckresource.Plate12
- pyhamilton.deckresource.Plate24
- pyhamilton.deckresource.Standard96

Class variables

Variable `align`

Variable `types`

Methods

Method `alignment_delta`

```
def alignment_delta(
    self,
    start,
    end
)
```

Method `layout_name`

```
def layout_name(
    self
)
```

The layout name of this specific deck resource.

Returns —-= The name (str) associated with this specific deck resource in the Hamilton Layout (.lay) file it came from.

Method `position_id`

```
def position_id(
    self,
    idx
)
```

The identifier used for one of a sequence of positions inside this labware.

For labware with multiple positions, each position has a different identity, usually represented as a short string that will match the identifier scheme for this resource in the Hamilton Layout file it came from. The identifiers will usually be familiar from a laboratory setting.

Examples

- 96-well plates have 96 positions, each identified with a letter and a number like `'D4'`. For a Plate96 instance named plate, plate.position_id(0) is `'A1'`, plate.position_id(1) is `'B1'`, and plate.position_id(95) is `'H12'`.

- Hamilton racks of 96 tips have 96 positions, identified with integer strings like `'87'` that start with `'1'` at the top left tip and increase down columns (8 positions each) first. For a Tip96 instance named tips, tips.position_id(0) is `'1'`, tips.position_id(1) is `'2'`, and tips.position_id(95) is `'96'`.

Args —-= **`idx`** : int : the index into the sequence of positions. Note: idx is zero-indexed across all labware according to python convention, while most real-world labware positions are 1-indexed.

Returns —-= The identifier (str) associated with the position idx specific deck resource in the Hamilton Layout (.lay) file it came from.

Raises —-= NotImplementedError : The deck resource does not have positions.

Class `LayoutManager`

```
class LayoutManager(
    layfile_path,
    install=True
)
```

Optionally activates a Hamilton layout and helps access its contents.

A LayoutManager manages the consistent assignment of DeckResource objects to items in a Hamilton Layout file (.lay). A LayoutManager must be used to set the active pyhamilton layout file, but use of this class is strictly optional when sending pyhamilton commands using send_command; names may be passed as string literals in commands instead if they are known in advance. The advantage to specifying all labware using ResourceManager is that resource names are verified to be present in the active layout file at runtime, and guaranteed never used more than once, both of which are necessary to avoid silent Hamilton errors.

Example usage:

```
lmgr = LayoutManager('layout.lay')
plate = lmgr.assign_unused_resource(ResourceType(Plate24, 'plate_0'))
culture_reservoir = lmgr.assign_unused_resource(ResourceType(Plate96, 'culture'))
inducer_tips = lmgr.assign_unused_resource(ResourceType(Tip96, 'inducer_tips'))
```

Static methods

Method `field_starts_with`

```
def field_starts_with(
    field,
    prefix
)
```

Method `get_manager`

```
def get_manager(
    checksum
)
```

Return a LayoutManager previously instantiated for a layout file that has the specified checksum.

Typically used when accessing the same layout file from multiple "threads" in the same process (using the threading module) to prevent name double-counting.

Args —-= **`checksum`** : str : a checksum found at the end of a Hamilton Layout (.lay) file.

Method initial_printable

```
def initial_printable(
    line,
    start=0
)
```

Method layfiles_equal

```
def layfiles_equal(
    lay_path_1,
    lay_path_2
)
```

Method layline_first_field

```
def layline_first_field(
    line
)
```

Method layline_objid

```
def layline_objid(
    line
)
```

Method line_has_prefixed_name

```
def line_has_prefixed_name(
    prefix
)
```

Method name_from_line

```
def name_from_line(
    line
)
```

Methods

Method `assign_unused_resource`

```
def assign_unused_resource(
    self,
    restype,
    order_key=None,
    reverse=False
)
```

Create a new deck resource after finding and assigning an unused name that matches the resource type.

This method searches through the layout file for one new layout name that matches the given resource type. It reserves this layout name permanently so that no later calls to assign_unused_resource can create a deck resource with the same layout name. Returns a DeckResource.

Args —-= **restype** : ResourceType : The resource type, which consists of a resource class (descendent of DeckResource) and some string pattern matching functions to identify the desired layout names.

`order_key` **: Callable[[DeckResource], Comparable]** Optional; when multiple layout names match, specifies a function of one argument that is used to extract a comparison key from each candidate DeckResource object. The arg-min or arg-max of order_key will be returned, depending on reverse. By default, lexicographic order by layout name is used, which is suitable for most use cases, e.g. plates with layout names “pcr-plate-a”, “pcr-plate-b”, “pcr-plate-c”, … will be returned in the expected order.

`reverse` **: bool** Optional; use reverse-lexicographic order for layout names, useful for e.g. plate stacking applications, or reverse the order imposed by order_key if it is given.

Returns —-= A new instance of the resource class (descendent of DeckResource) from the given ResourceType restype.

Raises —-= ResourceUnavailableError : no names in the layout file that have not already been assigned match the resource type

Class `Plate12`

```
class Plate12(
    layout_name
)
```

Ancestors (in MRO)

- pyhamilton.deckresource.DeckResource

Methods

Method `well_coords`

```
def well_coords(
    self,
    idx
)
```

Class `Plate24`

```
class Plate24(
    layout_name
)
```

Ancestors (in MRO)

- pyhamilton.deckresource.DeckResource

Methods

Method `well_coords`

```
def well_coords(
    self,
    idx
)
```

Class `Plate96`

```
class Plate96(
    layout_name
)
```

Labware types with 96 positions that use a letter-number id scheme like `'A1'`.

Ancestors (in MRO)

- pyhamilton.deckresource.Standard96
- pyhamilton.deckresource.DeckResource

Class `ResourceIterItem`

```
class ResourceIterItem(
    resource,
    index
)
```

Descendants

- pyhamilton.deckresource.Tip
- pyhamilton.deckresource.Vessel

Class `ResourceType`

```
class ResourceType(
    resource_class,
    *name_specifiers
)
```

Specifies a type of labware to extract using LayoutManager, and how.

This class associates a resource class, such as Plate96, with either a literal labware identifier (str) that appears in the Hamilton Layout (.lay) file, or a pair of functions: one that identifies when a text line in a layout file could be assigned this resource, called test, and one that parses such a name out of the line, called extract_name.

Typical usage:

```
plate_type = ResourceType(Plate96, 'Cos_96_Rd_0001')
lmgr = LayoutManager('layout.lay')
plate = lmgr.assign_unused_resource(plate_type)
```

Or:

```
plate_type = ResourceType(Plate96,
        LayoutManager.line_has_prefixed_name('Cos_96_Rd_'),
        LayoutManager.name_from_line)
lmgr = LayoutManager('layout.lay')
plate1 = lmgr.assign_unused_resource(plate_type)
plate2 = lmgr.assign_unused_resource(plate_type)
```

Args —-= **`resource_class`** : class : a class that inherits from DeckResource. Instances of this class will be returned from LayoutManager when assigning resources, factory-style.

`*name_specifiers` : list This argument is unpacked with the "splat" operator (*) to enable polymorphism. One or the other of:

- (two-argument form) an exact name (str) of a labware item that appears in the target layout file, or
- (three-argument form) test and extract_name (see usage above):

- test: a function [(`str`) -> bool] that identifies Layout file lines (str) that could be used to assign resources of this type
- extract_name: a function [(`str`) -> str] that gets the desired name out of a line identified with test.

Class `Standard96`

```
class Standard96(
    layout_name
)
```

Labware types with 96 positions that use a letter-number id scheme like `'A1'`.

Ancestors (in MRO)

- pyhamilton.deckresource.DeckResource

Descendants

- pyhamilton.deckresource.Plate96
- pyhamilton.deckresource.Tip96

Methods

Method `well_coords`

```
def well_coords(
    self,
    idx
)
```

Class `Tip`

```
class Tip(
    resource,
    index
)
```

Ancestors (in MRO)

- pyhamilton.deckresource.ResourceIterItem

Class `Tip96`

```
class Tip96(
    layout_name
)
```

Labware types with 96 positions that use a letter-number id scheme like `'A1'`.

Ancestors (in MRO)

- pyhamilton.deckresource.Standard96
- pyhamilton.deckresource.DeckResource

Class `Vessel`

```
class Vessel(
    resource,
    index
)
```

Ancestors (in MRO)

- pyhamilton.deckresource.ResourceIterItem

Class variables

Variable `ADD`

Variable `REMOVE`

Methods

Method `current_volume`

```
def current_volume(
    self
)
```

Method `record_addition`

```
def record_addition(
    self,
    ml,
    source
)
```

Method `record_removal`

```
def record_removal(
    self,
    ml,
    dest=None
)
```

Module `pyhamilton.defaultcmds`

Built-in commands, definitions of their parameters, and defaults.

Variables

Variable `defaults_by_cmd`

All of the command names supported out of the box, mapped to their default params.

On module load, defaults_by_cmd is parsed into HamiltonCmdTemplates, which are injected into the global package namespace under the first element of the values of this dict (strings in all caps). This is so that they can be imported directly from pyhamilton as module-level variables, while avoiding circular imports.

Example:

```
from pyhamilton import INITIALIZE
```

INITIALIZE

- initializeAlways (int)

  0=only initialize components not already initialized, 1=always reinitialize all robot components

  Default: 0

PICKUP

- tipSequence (string)

  leave empty if you are going to provide specific labwarePositions below

  Default: ’ ’

- labwarePositions (string)

  leave empty if you are going to provide a sequence name above.’LabwareId1, positionId1; LabwareId2,positionId2; ....’

  Default: ’ ’

- channelVariable (string)

  channel pattern e.g. ‘11110000’

  Default: _channel_patt_16

- sequenceCounting (integer)

  0=don´t autoincrement, 1=Autoincrement

  Default: 0

- channelUse (integer)

  1=use all sequence positions (no empty wells), 2=keep channel pattern

  Default: 1

EJECT

- wasteSequence (string)

  leave empty if you are going to provide specific labware-positions below or ejecting to default waste

  Default: ’ ’

- labwarePositions (string)

  leave empty if you are going to provide a sequence name above.’LabwareId1, positionId1; LabwareId2,positionId2; ....’

  Default: ’ ’

- channelVariable (string)

  channel pattern e.g. “11110000”

  Default: _channel_patt_16

- sequenceCounting (integer)

  0=don´t autoincrement, 1=Autoincrement. Value omitted if ejecting to default waste

  Default: 0

- channelUse (integer)

  1=use all sequence positions (no empty wells), 2=keep channel pattern

  Default: 1

- useDefaultWaste (integer)

  0=eject to custom waste sequence, 1=Use default waste

  Default: 0

ASPIRATE

- aspirateSequence (string)

  leave empty if you are going to provide specific labware-positions below

  Default: ’ ’

- labwarePositions (string)

  leave empty if you are going to provide a sequence name above. ‘LabwareId1, positionId1; LabwareId2,positionId2; ….’

  Default: ’ ’

- volumes (float or string)

  enter a single value used for all channels or enter an array of values for each channel like [10.0,15.5,11.2]

  Default: None

- channelVariable (string)

  channel pattern e.g. “11110000”

  Default: _channel_patt_16

- liquidClass (string)

  Default: None

- sequenceCounting (integer)

  0=don´t autoincrement, 1=Autoincrement

  Default: 0

- channelUse (integer)

  1=use all sequence positions (no empty wells), 2=keep channel pattern

  Default: 1

- aspirateMode (integer)

  0=Normal Aspiration, 1=Consecutive (don´t aspirate blowout), 2=Aspirate all

  Default: 0

- capacitiveLLD (integer)

  0=Off, 1=Max, 2=High, 3=Mid, 4=Low, 5=From labware definition

  Default: 0

- pressureLLD (integer)

  0=Off, 1=Max, 2=High, 3=Mid, 4=Low, 5=From liquid class definition

  Default: 0

- liquidFollowing (integer)

  0=Off , 1=On

  Default: 0

- submergeDepth (float)

  mm of immersion below liquid´s surface to start aspiration when using LLD

  Default: 2.0

- liquidHeight (float)

  mm above container´s bottom to start aspiration when not using LLD

  Default: 1.0

- maxLLdDifference (float)

  max mm height different between cLLD and pLLD detected liquid levels

  Default: 0.0

- mixCycles (integer)

  number of mixing cycles (1 cycle = 1 asp + 1 disp)

  Default: 0

- mixPosition (float)

  additional immersion mm below aspiration position to start mixing

  Default: 0.0

- mixVolume (float)

  mix volume

  Default: 0.0

- airTransportRetractDist (float)

  mm to move up in Z after finishing the aspiration at a fixed height before aspirating 'transport air'

  Default: 10.0

- touchOff (integer)

  0=Off , 1=On

  Default: 0

- aspPosAboveTouch (float)

  mm to move up in Z after touch off detects the bottom before aspirating liquid

  Default: 0.0

DISPENSE

- dispenseSequence (string)

  leave empty if you are going to provide specific labware-positions below

  Default: ' '

- labwarePositions (string)

  leave empty if you are going to provide a sequence name above. 'LabwareId1, positionId1; LabwareId2,positionId2; ....'

  Default: ' '

- volumes (float or string)

  enter a single value used for all channels or enter an array of values for each channel like [10.0,15.5,11.2]

  Default: None

- channelVariable (string)

  channel pattern e.g. “11110000”

  Default: _channel_patt_16

- liquidClass (string)

  Default: None

- sequenceCounting (integer)

  0=don´t autoincrement, 1=Autoincrement

  Default: 0

- channelUse (integer)

  1=use all sequence positions (no empty wells), 2=keep channel pattern

  Default: 1

- dispenseMode (integer)

  0=Jet Part, 1=Jet Empty, 2=Surface Part, 3=Surface Empty, 4=Jet Drain tip, 8=From liquid class, 9=Blowout tip

  Default: 8

- capacitiveLLD (integer)

  0=Off, 1=Max, 2=High, 3=Mid, 4=Low, 5=From labware definition

  Default: 0

- liquidFollowing (integer)

  0=Off , 1=On

  Default: 0

- submergeDepth (float)

  mm of immersion below liquid´s surface to start dispense when using LLD

  Default: 2.0

- liquidHeight (float)

  mm above container´s bottom to start dispense when not using LLD

  Default: 1.0

- mixCycles (integer)

  number of mixing cycles (1 cycle = 1 asp + 1 disp)

  Default: 0

- mixPosition (float)

  additional immersion mm below dispense position to start mixing

  Default: 0.0

- mixVolume (float)

  mix volume

  Default: 0.0

- airTransportRetractDist (float)

  mm to move up in Z after finishing the dispense at a fixed height before aspirating 'transport air'

  Default: 10.0

- touchOff (integer)

  0=Off , 1=On

  Default: 0

- dispPositionAboveTouch (float)

  mm to move up in Z after touch off detects the bottom, before dispense

  Default: 0.0

- zMoveAfterStep (integer)

  0=normal, 1=Minimized (Attention!!! this depends on labware clearance height, can crash).

  Default: 0

- sideTouch (integer)

  0=Off , 1=On

  Default: 0

PICKUP96

- tipSequence (string)

  leave empty if you are going to provide specific labware-positions below

  Default: ' '

- labwarePositions (string)

  leave empty if you are going to provide a sequence name above. 'LabwareId1, positionId1; LabwareId2,positionId2; ....' Must contain 96 values

  Default: ' '

- channelVariable (string)

  channel Variable e.g. "11110000...." . Must contain 96 values

  Default: _channel_patt_96

- sequenceCounting (integer)

  0=don´t autoincrement, 1=Autoincrement

  Default: 0

- reducedPatternMode (integer)

  0=All (not reduced), 1=One channel, 2=One row 3=One column

  Default: 0

EJECT96

- wasteSequence (string)

  leave empty if you are going to provide specific labware-positions below or ejecting to default waste

  Default: ' '

- labwarePositions (string)

  leave empty if you are going to provide a sequence name above. ‘LabwareId1, positionId1; LabwareId2,positionId2; ....’

  Default: ’ ’

- channelVariable (string)

  channel Variable e.g. “11110000....” . Must contain 96 values

  Default: _channel_patt_96

- sequenceCounting (integer)

  0=don´t autoincrement, 1=Autoincrement. Value omitted if ejecting to default waste

  Default: 0

- tipEjectToKnownPosition (integer)

  0=Eject to specified sequence position, 1=Eject on tip pick up position, 2=Eject on default waste

  Default: 0

ASPIRATE96

- aspirateSequence (string)

  leave empty if you are going to provide specific labware-positions below

  Default: ’ ’

- labwarePositions (string)

  leave empty if you are going to provide a sequence name above. LabwareId1, positionId1; LabwareId2,positionId2; ....

  Default: ’ ’

- aspirateVolume (float)

  single volume used for all channels in the head. There´s no individual control of each channel volume in multi-probe heads.

  Default: None

- channelVariable (string)

  channel Variable e.g. “11110000....” . Must contain 96 values

  Default: _channel_patt_96

- liquidClass (string)

  Default: None

- sequenceCounting (integer)

  0=don´t autoincrement, 1=Autoincrement

  Default: 0

- aspirateMode (integer)

  0=Normal Aspiration, 1=Consecutive (don´t aspirate blowout), 2=Aspirate all

  Default: 0

- capacitiveLLD (integer)

  0=Off, 1=Max, 2=High, 3=Mid, 4=Low, 5=From labware definition

  Default: 0

- liquidFollowing (integer)

  0=Off , 1=On

  Default: 0
- submergeDepth (float)

  mm of immersion below liquid´s surface to start aspiration when using LLD

  Default: 2.0
- liquidHeight (float)

  mm above container´s bottom to start aspiration when not using LLD

  Default: 1.0
- mixCycles (integer)

  number of mixing cycles (1 cycle = 1 asp + 1 disp)

  Default: 0
- mixPosition (float)

  additional immersion mm below aspiration position to start mixing

  Default: 0.0
- mixVolume (float)

  mix volume

  Default: 0.0
- airTransportRetractDist (float)

  mm to move up in Z after finishing the aspiration at a fixed height before aspirating 'transport air'

  Default: 10.0

DISPENSE96

- dispenseSequence (string)

  leave empty if you are going to provide specific labware-positions below

  Default: ' '
- labwarePositions (string)

  leave empty if you are going to provide a sequence name above. LabwareId1, positionId1; LabwareId2,positionId2; ....

  Default: ' '
- dispenseVolume (float)

  single volume used for all channels in the head. There´s no individual control of each channel volume in multi-probe heads.

  Default: None
- channelVariable (string)

  channel Variable e.g. "11110000...." . Must contain 96 values

  Default: _channel_patt_96
- liquidClass (string)

  Default: None

- sequenceCounting (integer)

  0=don´t autoincrement, 1=Autoincrement

  Default: 0

- dispenseMode (integer)

  0=Jet Part, 1=Jet Empty, 2=Surface Part, 3=Surface Empty,4=Jet Drain tip, 8=From liquid class, 9=Blowout tip

  Default: 8

- capacitiveLLD (integer)

  0=Off, 1=Max, 2=High, 3=Mid, 4=Low, 5=From labware definition

  Default: 0

- liquidFollowing (integer)

  0=Off , 1=On

  Default: 0

- submergeDepth (float)

  mm of immersion below liquid´s surface to start dispense when using LLD

  Default: 2.0

- liquidHeight (float)

  mm above container´s bottom to start dispense when not using LLD

  Default: 1.0

- mixCycles (integer)

  number of mixing cycles (1 cycle = 1 asp + 1 disp)

  Default: 0

- mixPosition (float)

  additional immersion mm below dispense position to start mixing

  Default: 0.0

- mixVolume (float)

  mix volume

  Default: 0.0

- airTransportRetractDist (float)

  mm to move up in Z after finishing the dispense at a fixed height before aspirating ‘transport air’

  Default: 10.0

- zMoveAfterStep (integer)

  0=normal, 1=Minimized (Attention!!! this depends on labware clearance height, can crash).

  Default: 0

- sideTouch (integer)

  0=Off , 1=On

  Default: 0

ISWAP_GET

plateSequence

`leave empty if you are going to provide specific plate labware-position below`

`Default:''`

- plateLabwarePositions (string)

  leave empty if you are going to provide a plate sequence name above. LabwareId1, positionId1;

  Default: ’’

- lidSequence (string)

  leave empty if you don´t use lid or if you are going to provide specific plate labware-positions below or ejecting to default waste

  Default: ’’

- lidLabwarePositions (string)

  leave empty if you are going to provide a plate sequence name above. LabwareId1, positionId1;

  Default: ’’

- toolSequence (string)

  sequence name of the iSWAP. leave empty if you are going to provide a plate sequence name above. LabwareId1, positionId1;

  Default: ’’

- sequenceCounting (integer)

  0=don´t autoincrement plate sequence, 1=Autoincrement

  Default: 0

- movementType (integer)

  0=To carrier, 1=Complex movement

  Default: 0

- transportMode (integer)

  0=Plate only, 1=Lid only ,2=Plate with lid

  Default: 0

- gripForce (integer)

  2 (minimum) ... 9 (maximum)

  Default: 4

- inverseGrip (integer)

  0=Off, 1=On

  Default: 0

- collisionControl (integer)

  0=Off, 1=On

  Default: 0

- gripMode (integer)

  0=Small side, 1=Large side

  Default: 1

- retractDistance (float)

  retract distance [mm] (only used if ‘movement type’ is set to ‘complex movement’)

  Default: 0.0

- liftUpHeight (float)

  lift-up distance [mm] (only used if ‘movement type’ is set to ‘complex movement’)

  Default: 20.0

- gripWidth (float)

  grip width when closed [mm]

  Default: 123.7

- tolerance (float)

  tolerance [mm]

  Default: 2.0

- gripHeight (float)

  height to grip above bottom of labware [mm]

  Default: 3.0

- widthBefore (float)

  grip width when opened before grip [mm]

  Default: 130.0

ISWAP_PLACE

- plateSequence (string)

  leave empty if you are going to provide specific plate labware-position below

  Default: ’ ’

- plateLabwarePositions (string)

  leave empty if you are going to provide a plate sequence name above. LabwareId1, positionId1;

  Default: ’ ’

- lidSequence (string)

  leave empty if you don´t use lid or if you are going to provide specific plate labware-positions below or ejecting to default waste

  Default: ’ ’

- lidLabwarePositions (string)

  leave empty if you are going to provide a plate sequence name above. LabwareId1, positionId1;

  Default: ’ ’

- toolSequence (string)

  sequence name of the iSWAP. leave empty if you are going to provide a plate sequence name above. LabwareId1, positionId1;

Default: ’ ’

- sequenceCounting (integer)

  0=don´t autoincrement plate sequence, 1=Autoincrement

  Default: 0

- movementType (integer)

  0=To carrier, 1=Complex movement

  Default: 0

- transportMode (integer)

  0=Plate only, 1=Lid only ,2=Plate with lid

  Default: 0

- collisionControl (integer)

  0=Off, 1=On

  Default: 0

- retractDistance (float)

  retract distance [mm] (only used if ‘movement type’ is set to ‘complex movement’)

  Default: 0.0

- liftUpHeight (float)

  lift-up distance [mm] (only used if ‘movement type’ is set to ‘complex movement’)

  Default: 20.0

HEPA

- deviceNumber (integer)

  COM port number of fan

  Default: _fan_port

- persistant (integer)

  0=don´t keep fan running after method exits, 1=keep settings after method exits

  Default: 1

- fanSpeed (float)

  set percent of maximum fan speed

  Default: None

- simulate (integer)

  0=normal mode, 1=use HxFan simulation mode

  Default: 0

WASH96_EMPTY

- refillAfterEmpty (integer)

  0=Don’t refill, 1=Refill both chambers, 2=Refill chamber 1 only, 3=Refill chamber 2 only

  Default: 0

- chamber1WashLiquid (integer)

  0=Liquid 1 (red container), 1=liquid 2 (blue container)

  Default: 0
- chamber1LiquidChange (integer)

  0=No, 1=Yes TODO: What does this mean?

  Default: 0
- chamber2WashLiquid (integer)

  0=Liquid 1 (red container), 1=liquid 2 (blue container)

  Default: 0
- chamber2LiquidChange (integer)

  0=No, 1=Yes TODO: What does this mean?

  Default: 0

Module `pyhamilton.interface`

Classes and utilities for automatic connection to a Hamilton robot.

Functions

Function `run_hamilton_process`

```
def run_hamilton_process()
```

Start the interpreter in a separate python process.

Starts the pyhamilton interpreter, which is an HSL file to be passed to the RunHSLExecutor.exe executable from Hamilton. This should always be done in a separate python process using the subprocess module, not a Thread.

Classes

Class `HamiltonCmdTemplate`

```
class HamiltonCmdTemplate(
    cmd_name,
    params_list
)
```

Formatter object to create valid pyhamilton command dicts.

Use of this class to assemble JSON pyhamilton commands enables keyword access to command attributes, which cuts down on string literals. It also helps to fail malformed commands early, before they are sent.

Several default HamiltonCmdTemplates are defined in pyhamilton.defaultcmds, such as INITIALIZE, ASPIRATE, and DISPENSE. Casual users will most likely never need to manually instantiate a HamiltonCmdTemplate.

Creates a HamiltonCmdTemplate with a command name and required parameters.

The command name must be one of the command names accepted by the pyhamilton interpreter and a list of expected parameters for this command.

Args —-= `cmd_name` : str : One of the set of string literals recognized as command names by the pyhamilton interpreter, e.g. `'mph96Dispense'`. See pyhamilton.defaultcmds for examples.

`params_list` : **list** exact list of string parameters that must have associated values for the command to be valid, other than those that are always present (`'command'` and `'id'`)

Static methods

`Method unique_id`

```
def unique_id()
```

Return a “uniqe” hexadecimal string (`'0x...'`) based on time of call.

Methods

Method `assemble_cmd`

```
def assemble_cmd(
    self,
    *args,
    **kwargs
)
```

Use keyword args to assemble this command. Default values auto-filled.

Args —-= `kwargs` : dict : map of any parameters (str) to values that should be different from the defaults supplied for this command in pyhamilton.defaultcmds

Method `assert_valid_cmd`

```
def assert_valid_cmd(
    self,
    cmd_dict
)
```

Validate a finished command. Do nothing if it is valid.

ValueError will be raised if the supplied command did not have all required parameters for this command, as well as values for keys `'id'` and `'command'`, which are always required.

Args —-= `cmd_dict` : dict : A fully assembled pyhamilton command

Raises —-= ValueError : The command dict is not ready to send. Specifics of mismatch summarized in exception description.

Class `HamiltonInterface`

```
class HamiltonInterface(
    address=None,
    port=None,
    simulate=False
)
```

Main class to automatically set up and tear down an interface to a Hamilton robot.

HamiltonInterface is the primary class offered by this module. It creates a Hamilton HSL background process running the pyhamilton interpreter, along with a localhost connection to act as a bridge. It is recommended to create a HamiltonInterface using a `with:` block to ensure proper startup and shutdown of its async components, even if exceptions are raised. It may be used with explicit start() and stop() calls.

Typical usage:

```
with HamiltonInterface() as ham_int:
    cmd_id = ham_int.send_command(INITIALIZE)
    ...
    ham_int.wait_on_response(cmd_id)
    ...
```

Class variables

Variable `HamiltonServerThread` Private threaded local HTTP server with graceful shutdown flag.

Variable `default_address`

Variable `default_port`

Variable `known_templates`

Methods

Method `is_open`

```
def is_open(
    self
)
```

Return True if the HamiltonInterface has been started and not stopped.

Method `log`

```
def log(
    self,
    msg,
    msg_type='info'
)
```

Method `log_and_raise`

```
def log_and_raise(
    self,
    err
)
```

Method `parse_hamilton_return`

```
def parse_hamilton_return(
    self,
    return_str
)
```

Return a 2-tuple:

- [0] errflag: any error code present in response
- [1] Block map: dict mapping int keys to dicts with str keys (MainErr, SlaveErr, RecoveryBtnId, StepData, LabwareName, LabwarePos)

Result value 3 is the field that is returned by the OEM interface.

“Result value 3 contains one error flag (ErrFlag) and the block data package.”

Data Block Format Rules

- The error flag is set once only at the beginning of result value 3. The error flag does not belong to the block data but may be used for a simpler error recovery. If this flag is set, an error code has been set in any of the block data entries.
- Each block data package starts with the opening square bracket character ‘[’
- The information within the block data package is separated by the comma delimiter ‘,’
- Block data information may be empty; anyway a comma delimiter is set.
- The result value may contain more than one block data package.
- Block data packages are returned independent of Num value (unsorted).

Block data information

- Num
  - Step depended information (e.g. the channel number, a loading position etc.).
  - Note: The meaning and data type for this information is described in the corresponding help of single step.
- MainErr
  - Main error code which occurred on instrument.
- SlaveErr
  - Detailed error code of depended slave (e.g. auto load, washer etc.).
- RecoveryBtnId
  - Recovery which has been used to handle this error.
- StepData
  - Step depended information, e.g. the barcode read, the volume aspirated etc.
  - Note: The meaning and data type for this information is described in the corresponding help of single step.
- LabwareName
  - Labware name of used labware.
- LabwarePos
  - Used labware position.

Method `pop_response`

```
def pop_response(
    self,
    id,
    raise_first_exception=False
)
```

Remove and return the response with the specified id from the response queue.

If there is a response, remove it and return the Hamilton-formatted response dict, like that returned from HamiltonInterface.parse_hamilton_return(). Otherwise, raise KeyError.

Args —-= `id` : str : Unique id of the command that initiated the response

`raise_first_exception` **: bool** Optional; forwarded to wait_on_response. Default is False.

Returns —-= A 2-tuple:

1. parsed response block dict from Hamilton as in parse_hamilton_return
2. Error map, a dict mapping int keys (data block Num field) that had exceptions, if any, to an exception that was coded in block; None to any error not associated with a block. `{}` if no error

Raises —-= KeyError : if id has no matching response in the queue.

Method `send_command`

```
def send_command(
    self,
    template=None,
    block_until_sent=False,
    *args,
    **cmd_dict
)
```

Add a command templated after HamiltonCmdTemplate to the server send queue.

Args —-= `template` : HamiltonCmdTemplate : Optional; a template to provide default arguments not specified in cmd_dict.

`block_until_sent` **: bool** Optional; if True, wait for all queued messages, including this one, to get picked up by the local server and sent across the HTTP connection, before returning. Default is False.

`cmd_dict` **: dict** keyword arguments to be forwarded to template when building the command, overriding its defaults. If template not given, cmd_dict must either have a 'command' key with value matching one of the command names in defaultcmds and might be missing an 'id' key, or itself be a fully formed and correct pyhamilton command with its own 'id' key.

Returns —-= unique id (str) of the command that can be used to index it later, either newly generated or same as originally present in cmd_dict.

Method `set_log_dir`

```
def set_log_dir(
    self,
    log_dir
)
```

Method `start`

```
def start(
    self
)
```

Starts the extra processes, threads, and servers for the Hamilton connection.

Launches: 1) the pyhamilton interpreter using the Hamilton Run Control executable, either in the background for normal use, or in the foreground with a GUI for simulation; 2) a local HTTP server to ferry messages between the python module and the interpreter.

When used with a `with:` block, called automatically upon entering the block.

Method `stop`

```
def stop(
    self
)
```

Stop this HamiltonInterface and clean up associated async processes.

Kills the pyhamilton interpreter subprocess and executable and stops the local web server thread.

When used with a with block, called automatically on exiting the block.

Method `wait_on_response`

```
def wait_on_response(
    self,
    id,
    timeout=0,
    raise_first_exception=False
)
```

Wait and do not return until the response for the specified id comes back.

When the command corresponding to id regards multiple distinct pipette channels or devices, responses may contain encoded errors that might be different for different channels or devices. For this reason, the default behavior of wait_on_response is to not raise exceptions, but to delegate handling exceptions to the caller. For convenience, this method can optionally raise the first exception it encounters, often a useful behavior for succinct scripted commands that regard only one device, when raise_first_exception is True.

Args —-= `id` : str : The unique id of a previously sent command

`timeout` **: float** Optional; maximum time in seconds to wait before raising HamiltonTimeoutError. Default is no timeout (forever).

`raise_first_exception` Optional; if True, may raise if there is an error encoded in the response. Default is False.

Returns —-= The response dict from the hamilton interpreter.

Raises —-= HamiltonTimeoutError: after timeout seconds elapse with no response, if timeout was specified.

Module `pyhamilton.oemerr`

pyhamilton-specific exception definitions.

Variables

Variable `HAMILTON_ERROR_MAP`

Maps integer error codes from Hamilton step return data to the appropriate pyhamilton errors

Classes

Class `AntiDropControlError`

```
class AntiDropControlError(
    *args,
    **kwargs
)
```

Anti drop controlling out of tolerance.

Ancestors (in MRO)

- pyhamilton.oemerr.HamiltonStepError
- pyhamilton.oemerr.HamiltonError
- builtins.Exception
- builtins.BaseException

Class `AreaAlreadyOccupiedError`

```
class AreaAlreadyOccupiedError(
    *args,
    **kwargs
)
```

Instrument region already reserved.

Ancestors (in MRO)

- pyhamilton.oemerr.HamiltonStepError
- pyhamilton.oemerr.HamiltonError
- builtins.Exception
- builtins.BaseException

Class `BarcodeAlreadyUsedError`

```
class BarcodeAlreadyUsedError(
    *args,
    **kwargs
)
```

The barcode read is already loaded as unique barcode (it’s not possible to load the same barcode twice).

Ancestors (in MRO)

- pyhamilton.oemerr.HamiltonStepError
- pyhamilton.oemerr.HamiltonError
- builtins.Exception
- builtins.BaseException

Class `BarcodeError`

```
class BarcodeError(
    *args,
    **kwargs
)
```

Barcode could not be read or is missing.

Ancestors (in MRO)

- pyhamilton.oemerr.HamiltonStepError
- pyhamilton.oemerr.HamiltonError
- builtins.Exception
- builtins.BaseException

Class `BarcodeMaskError`

```
class BarcodeMaskError(
    *args,
    **kwargs
)
```

The barcode read doesn’t match the barcode mask defined.

Ancestors (in MRO)

- pyhamilton.oemerr.HamiltonStepError
- pyhamilton.oemerr.HamiltonError
- builtins.Exception
- builtins.BaseException

Class `BarcodeNotUniqueError`

```
class BarcodeNotUniqueError(
    *args,
    **kwargs
)
```

The barcode read is not unique. Previously loaded labware with same barcode was loaded without unique barcode check.

Ancestors (in MRO)

- pyhamilton.oemerr.HamiltonStepError
- pyhamilton.oemerr.HamiltonError
- builtins.Exception
- builtins.BaseException

Class `CalibrateError`

```
class CalibrateError(
    *args,
    **kwargs
)
```

No capacitive signal detected during carrier calibration procedure.

Ancestors (in MRO)

- pyhamilton.oemerr.HamiltonStepError
- pyhamilton.oemerr.HamiltonError
- builtins.Exception
- builtins.BaseException

Class `ClotError`

```
class ClotError(
    *args,
    **kwargs
)
```

Blood clot detected.

Ancestors (in MRO)

- pyhamilton.oemerr.HamiltonStepError
- pyhamilton.oemerr.HamiltonError
- builtins.Exception
- builtins.BaseException

Class `CoverOpenError`

```
class CoverOpenError(
    *args,
    **kwargs
)
```

Cover not closed or can not be locked.

Ancestors (in MRO)

- pyhamilton.oemerr.HamiltonStepError
- pyhamilton.oemerr.HamiltonError
- builtins.Exception
- builtins.BaseException

Class `DecapperError`

```
class DecapperError(
    *args,
    **kwargs
)
```

Decapper lock error while screw / unscrew a cap by twister channels.

Ancestors (in MRO)

- pyhamilton.oemerr.HamiltonStepError
- pyhamilton.oemerr.HamiltonError
- builtins.Exception
- builtins.BaseException

Class `DecapperHandlingError`

```
class DecapperHandlingError(
    *args,
    **kwargs
)
```

Decapper station error while lock / unlock a cap.

Ancestors (in MRO)

- pyhamilton.oemerr.HamiltonStepError
- pyhamilton.oemerr.HamiltonError
- builtins.Exception
- builtins.BaseException

Class `DelimiterError`

```
class DelimiterError(
    *args,
    **kwargs
)
```

Barcode contains character which is used as delimiter in result string.

Ancestors (in MRO)

- pyhamilton.oemerr.HamiltonStepError
- pyhamilton.oemerr.HamiltonError
- builtins.Exception
- builtins.BaseException

Class `ExecutionError`

```
class ExecutionError(
    *args,
    **kwargs
)
```

A step or a part of a step could not be processed.

Ancestors (in MRO)

- pyhamilton.oemerr.HamiltonStepError
- pyhamilton.oemerr.HamiltonError
- builtins.Exception
- builtins.BaseException

Class `HamiltonDeckResourceError`

```
class HamiltonDeckResourceError(
    *args,
    **kwargs
)
```

Error with any deck object in interface with robot.

Ancestors (in MRO)

- pyhamilton.oemerr.HamiltonError
- builtins.Exception
- builtins.BaseException

Descendants

- pyhamilton.oemerr.ResourceUnavailableError

Class `HamiltonError`

```
class HamiltonError(
    *args,
    **kwargs
)
```

Exceptions raised in package pyhamilton

Ancestors (in MRO)

- builtins.Exception
- builtins.BaseException

Descendants

- pyhamilton.oemerr.HamiltonDeckResourceError
- pyhamilton.oemerr.HamiltonInterfaceError
- pyhamilton.oemerr.HamiltonStepError

Class `HamiltonInterfaceError`

```
class HamiltonInterfaceError(
    *args,
    **kwargs
)
```

Error in any phase of communication with robot.

Ancestors (in MRO)

- pyhamilton.oemerr.HamiltonError
- builtins.Exception
- builtins.BaseException

Descendants

- pyhamilton.oemerr.HamiltonReturnParseError
- pyhamilton.oemerr.HamiltonTimeoutError
- pyhamilton.oemerr.InvalidErrCodeError

Class `HamiltonReturnParseError`

```
class HamiltonReturnParseError(
    *args,
    **kwargs
)
```

Return string from instrument was malformed.

Ancestors (in MRO)

- pyhamilton.oemerr.HamiltonInterfaceError
- pyhamilton.oemerr.HamiltonError
- builtins.Exception
- builtins.BaseException

Class `HamiltonStepError`

```
class HamiltonStepError(
    *args,
    **kwargs
)
```

Errors in steps executed by VENUS software coded in the Hamilton error specification.

Ancestors (in MRO)

- pyhamilton.oemerr.HamiltonError
- builtins.Exception
- builtins.BaseException

Descendants

- pyhamilton.oemerr.AntiDropControlError
- pyhamilton.oemerr.AreaAlreadyOccupiedError
- pyhamilton.oemerr.BarcodeAlreadyUsedError
- pyhamilton.oemerr.BarcodeError
- pyhamilton.oemerr.BarcodeMaskError
- pyhamilton.oemerr.BarcodeNotUniqueError
- pyhamilton.oemerr.CalibrateError
- pyhamilton.oemerr.ClotError
- pyhamilton.oemerr.CoverOpenError
- pyhamilton.oemerr.DecapperError
- pyhamilton.oemerr.DecapperHandlingError
- pyhamilton.oemerr.DelimiterError
- pyhamilton.oemerr.ExecutionError
- pyhamilton.oemerr.HamiltonSyntaxError
- pyhamilton.oemerr.HardwareError
- pyhamilton.oemerr.IllegalInterventionError
- pyhamilton.oemerr.IllegalTargetPlatePositionError
- pyhamilton.oemerr.ImpossibleToOccupyAreaError
- pyhamilton.oemerr.ImproperAspirationOrDispenseError
- pyhamilton.oemerr.ImproperDispensationError
- pyhamilton.oemerr.InsufficientLiquidError
- pyhamilton.oemerr.KitLotExpiredError
- pyhamilton.oemerr.LabwareError
- pyhamilton.oemerr.LabwareGrippedError
- pyhamilton.oemerr.LabwareLostError
- pyhamilton.oemerr.LiquidLevelError
- pyhamilton.oemerr.NoCarrierBarcodeError
- pyhamilton.oemerr.NoCarrierError
- pyhamilton.oemerr.NoLabwareError
- pyhamilton.oemerr.NoTipError
- pyhamilton.oemerr.NotAspiratedError
- pyhamilton.oemerr.NotDetectedError
- pyhamilton.oemerr.NotExecutedError
- pyhamilton.oemerr.ParameterError
- pyhamilton.oemerr.PositionError
- pyhamilton.oemerr.PressureLLDError
- pyhamilton.oemerr.SlaveError
- pyhamilton.oemerr.TADMOvershotError
- pyhamilton.oemerr.TADMUndershotError
- pyhamilton.oemerr.TemperatureError
- pyhamilton.oemerr.TipPresentError
- pyhamilton.oemerr.UnexpectedLabwareError
- pyhamilton.oemerr.UnexpectedcLLDError
- pyhamilton.oemerr.UnloadError
- pyhamilton.oemerr.WashLiquidError
- pyhamilton.oemerr.WrongCarrierError
- pyhamilton.oemerr.WrongLabwareError

Class `HamiltonSyntaxError`

```
class HamiltonSyntaxError(
    *args,
    **kwargs
)
```

There is a wrong set of parameters or parameter ranges.

Ancestors (in MRO)

- pyhamilton.oemerr.HamiltonStepError
- pyhamilton.oemerr.HamiltonError
- builtins.Exception
- builtins.BaseException

Class `HamiltonTimeoutError`

```
class HamiltonTimeoutError(
    *args,
    **kwargs
)
```

An asynchronous request to the Hamilton robot timed out.

Ancestors (in MRO)

- pyhamilton.oemerr.HamiltonInterfaceError
- pyhamilton.oemerr.HamiltonError
- builtins.Exception
- builtins.BaseException

Class `HardwareError`

```
class HardwareError(
    *args,
    **kwargs
)
```

Steps lost on one or more hardware components, or component not initialized or not functioning.

Ancestors (in MRO)

- pyhamilton.oemerr.HamiltonStepError
- pyhamilton.oemerr.HamiltonError
- builtins.Exception
- builtins.BaseException

Class `IllegalInterventionError`

```
class IllegalInterventionError(
    *args,
    **kwargs
)
```

Cover was opened or a carrier was removed manually.

Ancestors (in MRO)

- pyhamilton.oemerr.HamiltonStepError
- pyhamilton.oemerr.HamiltonError
- builtins.Exception
- builtins.BaseException

Class `IllegalTargetPlatePositionError`

```
class IllegalTargetPlatePositionError(
    *args,
    **kwargs
)
```

Cannot place plate, plate was gripped in a wrong direction.

Ancestors (in MRO)

- pyhamilton.oemerr.HamiltonStepError
- pyhamilton.oemerr.HamiltonError
- builtins.Exception
- builtins.BaseException

Class `ImpossibleToOccupyAreaError`

```
class ImpossibleToOccupyAreaError(
    *args,
    **kwargs
)
```

A region on the instrument cannot be reserved.

Ancestors (in MRO)

- pyhamilton.oemerr.HamiltonStepError
- pyhamilton.oemerr.HamiltonError
- builtins.Exception
- builtins.BaseException

Class `ImproperAspirationOrDispenseError`

```
class ImproperAspirationOrDispenseError(
    *args,
    **kwargs
)
```

The pressure-based aspiration / dispensation control reported an error (not enough liquid).

Ancestors (in MRO)

- pyhamilton.oemerr.HamiltonStepError
- pyhamilton.oemerr.HamiltonError
- builtins.Exception
- builtins.BaseException

Class `ImproperDispensationError`

```
class ImproperDispensationError(
    *args,
    **kwargs
)
```

The dispensed volume is out of tolerance (may only occur for Nano Pipettor Dispense steps).

This error is created from main / slave error 02/52 and 02/54.

Ancestors (in MRO)

- pyhamilton.oemerr.HamiltonStepError
- pyhamilton.oemerr.HamiltonError
- builtins.Exception
- builtins.BaseException

Class `InsufficientLiquidError`

```
class InsufficientLiquidError(
    *args,
    **kwargs
)
```

Not enough liquid available.

Ancestors (in MRO)

- pyhamilton.oemerr.HamiltonStepError
- pyhamilton.oemerr.HamiltonError
- builtins.Exception
- builtins.BaseException

Class `InvalidErrCodeError`

```
class InvalidErrCodeError(
    *args,
    **kwargs
)
```

Error code returned from instrument not known.

Ancestors (in MRO)

- pyhamilton.oemerr.HamiltonInterfaceError
- pyhamilton.oemerr.HamiltonError
- builtins.Exception
- builtins.BaseException

Class `KitLotExpiredError`

```
class KitLotExpiredError(
    *args,
    **kwargs
)
```

Kit Lot expired.

Ancestors (in MRO)

- pyhamilton.oemerr.HamiltonStepError
- pyhamilton.oemerr.HamiltonError
- builtins.Exception
- builtins.BaseException

Class `LabwareError`

```
class LabwareError(
    *args,
    **kwargs
)
```

Labware not available.

Ancestors (in MRO)

- pyhamilton.oemerr.HamiltonStepError
- pyhamilton.oemerr.HamiltonError
- builtins.Exception
- builtins.BaseException

Class `LabwareGrippedError`

```
class LabwareGrippedError(
    *args,
    **kwargs
)
```

Labware already gripped.

Ancestors (in MRO)

- pyhamilton.oemerr.HamiltonStepError
- pyhamilton.oemerr.HamiltonError
- builtins.Exception
- builtins.BaseException

Class `LabwareLostError`

```
class LabwareLostError(
    *args,
    **kwargs
)
```

Labware lost during transport.

Ancestors (in MRO)

- pyhamilton.oemerr.HamiltonStepError
- pyhamilton.oemerr.HamiltonError
- builtins.Exception
- builtins.BaseException

Class `LiquidLevelError`

```
class LiquidLevelError(
    *args,
    **kwargs
)
```

Liquid surface not detected.

This error is created from main / slave error 06/70, 06/73 and 06/87.

Ancestors (in MRO)

- pyhamilton.oemerr.HamiltonStepError
- pyhamilton.oemerr.HamiltonError
- builtins.Exception
- builtins.BaseException

Class `NoCarrierBarcodeError`

```
class NoCarrierBarcodeError(
    *args,
    **kwargs
)
```

Carrier barcode could not be read or is missing.

Ancestors (in MRO)

- pyhamilton.oemerr.HamiltonStepError
- pyhamilton.oemerr.HamiltonError
- builtins.Exception
- builtins.BaseException

Class `NoCarrierError`

```
class NoCarrierError(
    *args,
    **kwargs
)
```

No carrier present for loading.

Ancestors (in MRO)

- pyhamilton.oemerr.HamiltonStepError
- pyhamilton.oemerr.HamiltonError
- builtins.Exception
- builtins.BaseException

Class `NoLabwareError`

```
class NoLabwareError(
    *args,
    **kwargs
)
```

The labware to be loaded was not detected by autoload module.

Note:

May only occur on a Reload Carrier step if the labware property 'MlStarCarPosAreRecognizable' is set to 1.

Ancestors (in MRO)

- pyhamilton.oemerr.HamiltonStepError
- pyhamilton.oemerr.HamiltonError
- builtins.Exception
- builtins.BaseException

Class `NoTipError`

```
class NoTipError(
    *args,
    **kwargs
)
```

Tip is missing or not picked up.

Ancestors (in MRO)

- pyhamilton.oemerr.HamiltonStepError
- pyhamilton.oemerr.HamiltonError
- builtins.Exception
- builtins.BaseException

Class `NotAspiratedError`

```
class NotAspiratedError(
    *args,
    **kwargs
)
```

Dispense volume exceeds the aspirated volume.

This error is created from main / slave error 02/54.

Ancestors (in MRO)

- pyhamilton.oemerr.HamiltonStepError
- pyhamilton.oemerr.HamiltonError
- builtins.Exception
- builtins.BaseException

Class `NotDetectedError`

```
class NotDetectedError(
    *args,
    **kwargs
)
```

Carrier not detected at deck end position.

Ancestors (in MRO)

- pyhamilton.oemerr.HamiltonStepError
- pyhamilton.oemerr.HamiltonError
- builtins.Exception
- builtins.BaseException

Class `NotExecutedError`

```
class NotExecutedError(
    *args,
    **kwargs
)
```

There was an error in previous part command.

Ancestors (in MRO)

- pyhamilton.oemerr.HamiltonStepError
- pyhamilton.oemerr.HamiltonError
- builtins.Exception
- builtins.BaseException

Class `ParameterError`

```
class ParameterError(
    *args,
    **kwargs
)
```

Dispense in jet mode with pressure liquid level detection is not allowed.

Ancestors (in MRO)

- pyhamilton.oemerr.HamiltonStepError
- pyhamilton.oemerr.HamiltonError
- builtins.Exception
- builtins.BaseException

Class `PositionError`

```
class PositionError(
    *args,
    **kwargs
)
```

The position is out of range.

Ancestors (in MRO)

- pyhamilton.oemerr.HamiltonStepError
- pyhamilton.oemerr.HamiltonError
- builtins.Exception
- builtins.BaseException

Class `PressureLLDError`

```
class PressureLLDError(
    *args,
    **kwargs
)
```

Pressure liquid level detection in a consecutive aspiration is not allowed.

Ancestors (in MRO)

- pyhamilton.oemerr.HamiltonStepError
- pyhamilton.oemerr.HamiltonError
- builtins.Exception
- builtins.BaseException

Class `ResourceUnavailableError`

```
class ResourceUnavailableError(
    *args,
    **kwargs
)
```

Layout manager found deck resource type not present or all of this type assigned

Ancestors (in MRO)

- pyhamilton.oemerr.HamiltonDeckResourceError
- pyhamilton.oemerr.HamiltonError
- builtins.Exception
- builtins.BaseException

Class `SlaveError`

```
class SlaveError(
    *args,
    **kwargs
)
```

Slave error.

Ancestors (in MRO)

- pyhamilton.oemerr.HamiltonStepError
- pyhamilton.oemerr.HamiltonError
- builtins.Exception
- builtins.BaseException

Class `TADMOvershotError`

```
class TADMOvershotError(
    *args,
    **kwargs
)
```

Overshot of limits during aspirate or dispense.

Note:

On aspirate this error is returned as main error 17.

On dispense this error is returned as main error 4.

Ancestors (in MRO)

- pyhamilton.oemerr.HamiltonStepError
- pyhamilton.oemerr.HamiltonError
- builtins.Exception
- builtins.BaseException

Class `TADMUndershotError`

```
class TADMUndershotError(
    *args,
    **kwargs
)
```

Undershot of limits during aspirate or dispense.

Note:

On aspirate this error is returned as main error 4.

On dispense this error is returned as main error 17.

Ancestors (in MRO)

- pyhamilton.oemerr.HamiltonStepError
- pyhamilton.oemerr.HamiltonError
- builtins.Exception
- builtins.BaseException

Class `TemperatureError`

```
class TemperatureError(
    *args,
    **kwargs
)
```

Incubator temperature out of range.

Ancestors (in MRO)

- pyhamilton.oemerr.HamiltonStepError
- pyhamilton.oemerr.HamiltonError
- builtins.Exception
- builtins.BaseException

Class `TipPresentError`

```
class TipPresentError(
    *args,
    **kwargs
)
```

A tip has already been picked up.

Ancestors (in MRO)

- pyhamilton.oemerr.HamiltonStepError
- pyhamilton.oemerr.HamiltonError
- builtins.Exception
- builtins.BaseException

Class `UnexpectedLabwareError`

```
class UnexpectedLabwareError(
    *args,
    **kwargs
)
```

The labware contains unexpected barcode (may only occur on a Reload Carrier step).

Ancestors (in MRO)

- pyhamilton.oemerr.HamiltonStepError
- pyhamilton.oemerr.HamiltonError
- builtins.Exception
- builtins.BaseException

Class `UnexpectedcLLDError`

```
class UnexpectedcLLDError(
    *args,
    **kwargs
)
```

The cLLD detected a liquid level above start height of liquid level search.

Ancestors (in MRO)

- pyhamilton.oemerr.HamiltonStepError
- pyhamilton.oemerr.HamiltonError
- builtins.Exception
- builtins.BaseException

Class `UnloadError`

```
class UnloadError(
    *args,
    **kwargs
)
```

Not possible to unload the carrier due to occupied loading tray position.

Ancestors (in MRO)

- pyhamilton.oemerr.HamiltonStepError
- pyhamilton.oemerr.HamiltonError
- builtins.Exception
- builtins.BaseException

Class `WashLiquidError`

```
class WashLiquidError(
    *args,
    **kwargs
)
```

Waste full or no more wash liquid available.

Ancestors (in MRO)

- pyhamilton.oemerr.HamiltonStepError
- pyhamilton.oemerr.HamiltonError
- builtins.Exception
- builtins.BaseException

Class `WrongCarrierError`

```
class WrongCarrierError(
    *args,
    **kwargs
)
```

Wrong carrier barcode detected.

Ancestors (in MRO)

- pyhamilton.oemerr.HamiltonStepError
- pyhamilton.oemerr.HamiltonError
- builtins.Exception
- builtins.BaseException

Class `WrongLabwareError`

```
class WrongLabwareError(
    *args,
    **kwargs
)
```

The labware to be reloaded contains wrong barcode (may only occur on a Reload Carrier step).

Ancestors (in MRO)

- pyhamilton.oemerr.HamiltonStepError
- pyhamilton.oemerr.HamiltonError
- builtins.Exception
- builtins.BaseException

Module `pyhamilton.util`

Classes

Class `ChannelHead`

```
class ChannelHead
```

Descendants

- pyhamilton.util.Independent8Channel
- pyhamilton.util.Standard96Channel

Methods

Method `can_move_simul`

```
def can_move_simul(
    move1,
    move2
)
```

Method `pack_moves`

```
def pack_moves(
    self,
    moves
)
```

Class `FlyTransfer`

```
class FlyTransfer(
    tip,
    ml,
    source,
    dest
)
```

Ancestors (in MRO)

- pyhamilton.util.Transfer
- pyhamilton.util.GroupableAction
- pyhamilton.util.HamiltonAction

Class `GroupableAction`

```
class GroupableAction
```

Ancestors (in MRO)

- pyhamilton.util.HamiltonAction

Descendants

- pyhamilton.util.TipPickup
- pyhamilton.util.Transfer

Class `HamiltonAction`

```
class HamiltonAction
```

Descendants

- pyhamilton.util.GroupableAction
- pyhamilton.util.Mix

Methods

Method `execute`

```
def execute(
    self
)
```

Method `possible`

```
def possible(
    self
)
```

Class `HamiltonCoordinator`

```
class HamiltonCoordinator(
    hamilton_interface,
    channel_heads
)
```

Methods

Method `execute`

```
def execute(
    self,
    specific_actions=None
)
```

Method `stage`

```
def stage(
    self,
    action
)
```

Method `wait_for_all`

```
def wait_for_all()
```

Class `HamiltonDevice`

```
class HamiltonDevice(
    interface,
    heads
)
```

Class `Independent8Channel`

```
class Independent8Channel
```

Ancestors (in MRO)

- pyhamilton.util.ChannelHead

Class `Mix`

```
class Mix(
    tip,
    ml,
    target,
    times
)
```

Ancestors (in MRO)

- pyhamilton.util.HamiltonAction

Class `Standard96Channel`

```
class Standard96Channel
```

Ancestors (in MRO)

- pyhamilton.util.ChannelHead

Class `TipPickup`

```
class TipPickup(
    tip
)
```

Ancestors (in MRO)

- pyhamilton.util.GroupableAction
- pyhamilton.util.HamiltonAction

Methods

Method `execute`

```
def execute(
    self
)
```

Class `Transfer`

```
class Transfer(
    tip,
    ml,
    source,
    dest
)
```

Ancestors (in MRO)

- pyhamilton.util.GroupableAction
- pyhamilton.util.HamiltonAction

Descendants

- pyhamilton.util.FlyTransfer

Methods

Method `execute`

```
def execute(
    self
)
```

Generated by *pdoc* 0.9.1 (https://pdoc3.github.io).
